# Management of the Pedicle in Ovarian Cystomata

**Published:** 1891-04

**Authors:** A. C. Walker

**Affiliations:** Rockdale, Texas


For Daniel's Texas Medical Journal.

### MANAGEMENT OF PEDICLE IN OVARIAN CYSTOMATA.

A. C. WALKER, M. D., ROCKDALE, TEXAS.

OPERATIONS for the removal of ovarian cysts, though the tumor be enormous in its proportions and environed with complications innumerable, are of such acceptable frequency in this surgical era of ours, that the report of a successful result could but record one more to the already scores which have appeared in our medical journals for years past.

But when in our surgical course we find that certain procedures in technique fulfill the indications for which they are aimed and furnish conclusions, which to our minds, are fruitful in true worth, then to the profession do we owe such report and respectfully ask a just consideration.

Much has been written, and various have been the opinions of operators upon the method of treating the pedicle in ovariotomy. However, I wish only to call attention to one point in its management, which I believe has served well in one instance.

### CASE.

Mrs. B., age 42; married; no children; menstruation irregular; feeble and emaciated; tumor had been noticeable for years back; Nov. 19th, 1890, made ovariotomy. Medium abdominal incision six inches in length; tumor polycystic in character; contents readily evacuated with Emmet's ovarian trocar. The attachments were extensive, requiring many ligatures while separating the bands between the omentum and abdominal viscera. After much careful work in the way of extricating the sac which was wedged and firmly bound down in the pelvis cavity, the entire ovarian sac was extruded through the abdominal incision. Observing all the antiseptic precautions incident to this stage of the operation, diligent search was made for pedicle attachment, which was located on the right side. Instead of a true narrow pedicle, as had been my good fortune to find in previous operations, there existed no pedicle, a contraction of the cyst wall, including the ovarian ligament, fallopian tube and broad ligament was the nature of the pedicle to deal with. This growth arose from or extended to the lateral border of the fundus uteri. So intricate was the connection of uterus and ovarian sac, that in tying the mass it was necessary to bring the ligatures within one-half inch of the uterine body.

The pseudo-pedicle was ligated with No. — antiseptic braided silk, the width of the cysto-uterine attachment being so great as to require the dividing of it into three sections, after which the tumor was severed about one and a half inches from ligature.

The gaping mouth of the exposed stump gave the appearance of a leather sack with a string tied near its end, showing conclusively to me that I had ligated or closed the sac itself and not tied off an impervious pedicle, as is the custom to find in such cases. The gravity of the attachments in addition to this huge exposed sac stump gave an ominous outlook for my patient. I apprehended subsequent septic infection from the sloughing of a pedicle of its proportions. While in a quandary what to do, I discovered that the investing peritoneum had been stripped from the tumor in my effort to pedunculate the mass, and that it had not been included in the pedicle ligation, I at once decided to utilize this valuable membrane to render the stump and the puckered hollow extremity perfectly aseptic, to cover and hermetically seal this exposed end with the thickened cyst peritonem, which I did by suturing with fine cat gut, using the continuous suture thus closing from view the stump end. The silk pedicle ligatures were cut short and the stump, or rather the uterus dropped back in the pelvic cavity. The rules attending the peritoneal toilette and closing the abdominal wound were scrupulously observed as in like operations.

With but a few disturbances, the patient went on to a favorable termination ; and now at this writing is well, attending to her usual duties. It is my conviction that the manner in which I treated the stump contributed no little towards the recovery of my patient.

The tumor was large—weight 50 lbs.; the attachments were extensive ; hemorrhage was considerable, and finally a tumor without pedicle, therefore requiring an unusual degree of force in ligating off the sac, as well as the undue energy exercised in extruding the tumor while separating its attachments. With these complications, I could but feel that a subsidiary evil, as a sloughing or septic stump, would be disastrous to my patient.

It is remarkable to suppose that a large ovarian stump, created from the sac itself, its distal end gathered in folds which of necessity formed crevices and cavities numberless, would be a convenient receptacle for retention of matter and to consequent septic changes. On the other hand, I contend that it is rational, that when this stump and its stump cavity is rendered perfectly aseptic, trimmed of redundant tissue, neatly and securely covered with a vascular membrane such as we find in the peritoneum, that the adhesive inflammation which occurs will be rapid in progress, the plastic exudate speedily organize, the formative cells develop new blood vessels, which in turn will supply tissue nutriment to the strangulated extremity, presenting in due time a stump possessed of independent and vital support.

